# Supplementary material for: C-reactive protein and risk of cognitive decline: The REGARDS study
Source: PLoS One. 2020 Dec 31;15(12):e0244612. doi: 10.1371/journal.pone.0244612 (PMC7774911; doi:10.1371/journal.pone.0244612)
Supplement: Attachment — Submitted filename: CRP PlosOne_2nd Response to Reviewers.docx [file pone.0244612.s002.docx]

**PLOS ONE MS ID#:** PONE-D-20-23675

**MS TITLE:** C-reactive protein and risk of cognitive decline: The REGARDS study

Dear Dr. Ginsberg,

We greatly appreciate the careful second review of our manuscript and are delighted that you will consider a revised version for publication. We have included a tracked changes version of the manuscript with changed text highlighted in yellow for convenience. We hope that our changes have adequately addressed the suggestions and that the revision will be suitable for publication in PLOS ONE.

**Reviewer #2:** The quality of the manuscript was improved. Please find some minor comments below.

1. Line 74. The word “in” appears twice. Please delete one.

We have removed this typo.

2. Please include in the limitations of the study the fact that weight and height were not measured in duplicate.

While we agree it would be ideal to measure everything in duplicate, due to the inherent design of a large epidemiological study such as REGARDS, it is not feasible to accomplish that. We are not concerned that this lack of duplicate measurement for weight and height is a notable limitation to the aims of our particular study. While lack of a duplicate height and weight measurement may lead to measurement error in a confounder (BMI), we are able to overcome this potential measurement error due to the statistical power provided by our large sample size. Moreover, this potential issue would lead to BMI further attenuating the relationship of CRP with our cognitive outcomes thus biasing to the null hypothesis. Regardless, we have added the following statement in our limitations section:

“Another similar potential limitation is that we only measured several covariates (i.e., height and weight) at baseline. Like most epidemiologic studies, for reasons of pragmatism, all baseline variables cannot be measured twice, which would improve precision of risk estimates. The impact of this limitation would be a bias to the null hypothesis.”

3. There are still several sentences using “greater”, for which “higher” would be more appropriate.

We have removed the word “greater” throughout the manuscript and updated it for “higher” or “increase” where appropriate.

4. There are still some sentences in which the word "effect" must be avoided, because of describing cross-sectional findings (example: line 241).

We have switched “effect” for “association” as appropriate throughout the manuscript.

5. Line 267-269: Authors say that “recent studies have reported (…) greater risk of dementia in later life [6]”. However, reference 6 is not “recent”, since it was published in 2002.

We removed “recent” from that sentence.

Lastly, an additional comment to address was that Reviewer #2 answered “No” to the following question “Have the authors made all data underlying the findings in their manuscript fully available?” and we would just like to reiterate that we addressed this in our previous cover letter as suggested by editor with the following information:

“The data underlying the findings include potentially identifying participant information and cannot be made publicly available due to ethical/legal restrictions. However, data including statistical code from this manuscript are available to researchers who meet the criteria for access to confidential data. Data can be obtained upon request through the University of Alabama at Birmingham at [regardsadmin@uab.edu](mailto:regardsadmin@uab.edu).”
